# Identifying and quantifying potential super-spreaders in social networks

**DOI:** 10.1038/s41598-019-51153-5

**Published:** 2019-10-15

**Authors:** Dayong Zhang, Yang Wang, Zhaoxin Zhang

**Affiliations:** 1 Department of New Media and Arts, Harbin Institute of Technology, Harbin, 150001 China; 2 School of Computer Science and Technology, Harbin Institute of Technology, Weihai, 264209 China

**Keywords:** Information technology, Complex networks

## Abstract

Quantifying the nodal spreading abilities and identifying the potential influential spreaders has been one of the most engaging topics recently, which is essential and beneficial to facilitate information flow and ensure the stabilization operations of social networks. However, most of the existing algorithms just consider a fundamental quantification through combining a certain attribute of the nodes to measure the nodes’ importance. Moreover, reaching a balance between the accuracy and the simplicity of these algorithms is difficult. In order to accurately identify the potential super-spreaders, the CumulativeRank algorithm is proposed in the present study. This algorithm combines the local and global performances of nodes for measuring the nodal spreading abilities. In local performances, the proposed algorithm considers both the direct influence from the node’s neighbourhoods and the indirect influence from the nearest and the next nearest neighbours. On the other hand, in the global performances, the concept of the tenacity is introduced to assess the node’s prominent position in maintaining the network connectivity. Extensive experiments carried out with the Susceptible-Infected-Recovered (SIR) model on real-world social networks demonstrate the accuracy and stability of the proposed algorithm. Furthermore, the comparison of the proposed algorithm with the existing well-known algorithms shows that the proposed algorithm has lower time complexity and can be applicable to large-scale networks.

## Introduction

Spreading process is a ubiquitous phenomenon in the nature^1,2^. In most of the real-world networks, the spreading process is the results of the interactions between infected individuals and uninfected individuals. Some spreading processes, including virus propagation and rumor spreading have profound negative economical and social impacts^3,4^. In order to prevent potential disruptions and ensure social stability, external intervention is widely essential as the most effective way to control epidemic transmission or rumor spreading^5,6^. Specifically, influential users, which are source spreaders, can obtain higher information diffusion levels on the network than non-influential users^7^. Thus, the optimized solution for the influence maximization problem is the most common and effective intervention method. However, quantifying the individual spreading abilities in complex networks, especially identifying the potential super-spreaders in a large-scale social network, is still a big challenge nowadays^8–11^.

The term “super-spreaders” refers to those who are particularly effective in transmitting infectious diseases or spreading information. In epidemiology, a super-spreader is an infected organism that infects disproportionally more secondary contacts than others who are also infected with the same disease^12^. Similarly, in information diffusion, super-spreaders play a more important role than normal individuals in promoting the information diffusion or determining the emergence of hot topics, including opinion leaders who have the ability to influence others to share and retweet their messages^13^, information brokers who connect different groups of users or have strong ties with the influential followers^14^. In fact, identifying individuals with the ability to be super-spreaders during virus propagation and rumor spreading can help us to better prevent the epidemic or public events bursting^15,16^.

The ranking algorithms were proposed initially to investigate the influence or prestige of individuals in social networks by several social scientists^17,18^. Nowadays, the ranking algorithms are introduced to study real-world issues. Moreover, they are utilized for novel applications, including optimizing communication networks^19^, finding social leaders^20^, and assessing network vulnerability^21^. However, these conventional centrality indices just consider a fundamental quantification through combing geodesics between individuals and they are not suitable to describe the pivotal positions of individuals from multiple angles. Thus, some researchers have attempted to redefine the concept of the individual influence. Stephenson and Zelen introduced the concept of the information centrality to capture the information contained in all possible paths of a connected network^22^. Kitsak *et al*.proposed the k-shell decomposition algorithm to evaluate the nodal spreading capabilities^8^. Wang and Zhao suggested a multi-attribute integrated index based on the degree centrality, the closeness centrality, the clustering coefficient and the topology potential^23^. Sheikhahmadi and Nematbakhsh introduced a hybrid algorithm called the MCDE algorithm^16^. Ahajjam and Badir provided a new centrality for the identification of influential nodes in networks based on the improved coreness centrality and the eigenvector centrality^7^.

Furthermore, with the explosive data growth, designing efficient and effective ranking algorithms on large-scale networks has attracted considerable attentions. Some diffusion algorithms based on random-walk were proposed, including the well-known PageRank^24^, HITS scores^25^, LeaderRank^20^, ClusterRank^26^ and other improved PageRank algorithms^27,28^. These representative algorithms have a common assumption that a node is expected to be of high influence if it points to many highly influential neighbours. So, they work well for directed networks, however they have a poor performance for undirected networks.

In fact, many researches regarding artificial network dataset and real social networks have demonstrated that when investigating the effectiveness of a ranking algorithm, it must be combined with the structural properties of networks and a certain functional goal^29,30^. For example, identifying influential nodes according to their roles in maintaining the network connectivity or facilitating information flow^31^. In addition, the existing ranking algorithms are difficult to reach a balance between accuracy and simplicity. In other words, some measures perform very simple but limit the accuracy, such as the local centrality indices, whereas others with a high computational complexity perform accurately but are incapable to be applied in large-scale networks, such as the global centrality indices. Thus, in the present study it is intended to fill this gap by exploring a new algorithm to quantify the nodal spreading abilities and identify potential super-spreaders in real-world social networks.

In the present study, a new ranking algorithm named CumulativeRank is proposed to quantify the nodal spreading abilities, which combines the local and global performances of each node in a given social network. Finally, the SIR model is applied to simulate the spreading processes in real social networks. The experimental results show that the proposed algorithm outperforms other well-known ranking algorithms in terms of accuracy and simplicity. The contributions of this study can be summarized as the following:

- The CumulativeRank algorithm is a three-step implementation algorithm that combined the local and global performances of each node.
- The improved network constraint coefficient (INCC) is proposed to assess the local performances of each node.
- The concept of the tenacity is introduced to measure the node’s prominent position in maintaining the network’s connectivity.
- The experimental results verify the outperformance of the proposed algorithm in terms of accuracy and simplicity.

The rest of the paper is organized as the following: In section 2, several widely-used ranking algorithms are reviewed briefly and the SIR model is introduced as the evaluation metric. In Section 3, the proposed algorithm is described in detail. In Section 4, the SIR model is applied to compare the performances of the proposed algorithm and six well-known ranking algorithms in real-world datasets. Finally, conclusions are given in Section 5.

## Background

### Ranking algorithms

A social network is a social structure made up of social members and their relationships. From the view of graph theory, most of social networks can be written as a graph *G* = (*V*, *E*), where $V=\{{v}_{1},{v}_{2},\cdots \cdots \,{v}_{n}\}$ represents a node set and $E=\{{e}_{1},{e}_{2},\cdots \cdots \,{e}_{m}\}$ represents an edge set. A node *v*_*i*_ ∈ *V* denotes an individual or organizations in the social network. Moreover, an edge *e*_*i*_ ∈ *E* denotes a possible social interaction between individuals or organizations, including communication or collaboration between members of a social group. The number of elements in *V* and *E* are presented by n and m, respectively. Nowadays, with the emergence of web2.0, people can share their opinions, communicate and relate to one another anytime and anywhere. In fact, some individuals play an important role in facilitating information flow and ensuring the stabilization operations of the whole network. In this section, five widely-used ranking algorithms, including degree centrality, betweenness centrality, eigenvector centrality, LocalRank, PageRank and a hybrid algorithm defined by Fu *et al*.^32^ are introduced as benchmark algorithms.

#### Degree centrality (DC)

The DC is obtained by calculating the ratio of the number of edges of node *i* to the maximum possible number of edges, which reflects the ability of a node to connect directly with other nodes.

#### Betweenness centrality (BC)

The BC measures a node’s influence through the ratio of the shortest path over the nodes to the number of all paths. The BC considers the global structure information of a given graph. The higher the BC value of a node, the stronger its controlling or spreading abilities.

#### Eigenvector centrality (EC)

Another important index in the category of global centrality indices is the EC, which considers that the centrality of a node depends not only on the number of its neighbours, but also on the centrality of its neighbours.

#### LocalRank (LR)

Most of the global centrality indices have rather high computational complexity, which restricts their applications to large-scale networks. To overcome this problem, Chen *et al*.^33^ proposed a semi-local centrality, named the LocalRank, as a tradeoff between the local centrality indices and the global centrality indices. The LR value of node *i* is written as the following:

1 $$L{R}_{i}=\sum _{j\in \varGamma (i)}{Q}_{j}$$

2 $${Q}_{j}=\sum _{w\in \varGamma (j)}{N}_{w}$$

where Γ(*i*) and *N*_(*w*)_ are the total number of first-degree neighbours of the node *i* and the total number of first- and second-degree neighbours of node *w*, respectively.

#### PageRank (PR)

The PR is an application of the random walker model on a Markov chain^24^, where nodes are the web pages and edges represent the links from one page to another. The PR value of a page *i* at *t* step is described as the following:

3 $$P{R}_{i}^{t}=a\sum _{j\in {\varGamma }_{i}^{in}}\frac{P{R}^{t-1}(j)}{{k}_{j}^{out}}+\frac{1-a}{n}$$

Where the damping factor *a* ∈ [0, 1] is usually set to be around 0.85. Moreover, ${\Gamma }_{i}^{in}$, ${k}_{j}^{out}$ and *n* are the set of pages that link to page *i*, the out-degree of page *j* and is the total number of pages, respectively.

#### Fu *et al*.’ algorithm (FA)

Fu *et al*.^32^ proposed a hybrid algorithm that combines global diversity and local features to identify the most influential network nodes. They used the k-shell entropy to measure the global connecting capability of each node and considered the local degree values of its neighbours. The FA value of node *i* is defined as the following:

4 $$F{A}_{i}={E}_{i}\cdot {L}_{i}$$

Where *E*_*i*_ and *L*_*i*_ are the k-shell entropy value of node *i* and the sum of all neighbours’ degree centrality values within the two-step distance of node *i*, respectively.

### SIR model

Centrality measures provide a method to quantify the nodal influence values, however the numeric values may not be directly interpretable^34^. As the information diffusion is similar to the epidemic spreading, a series of epidemic models are proposed to track the information spreading process and identify the influential spreaders. In the present study, the standard SIR model is utilized to estimate the spreading abilities of selected individuals and illustrate competitive advantages of the proposed algorithm over the conventional well-known algorithms. It should be noted that the standard SIR model assumes that nodes in a network can be in one of three possible states, including susceptible (denoted by S), infected (denoted by I) and recovered (denoted by R). Only a few individuals are set to be infective initially, while other individuals are in the susceptible state. The initial infected individuals are the originators of diseases, which can be obtained by various ranking indices. Once susceptible nodes get in contact with one or more infected neighbours, they become infected with the infection probability of *β*. Meanwhile, the infected individuals can be cured with the recovery probability of *γ*. The epidemic spreading is repeated until there are no infected individuals in the network and the network reaches a stable state. Since the large infection probability *β* makes the spreading cover almost all of the network, where the role of the individual is no longer important, the *β* value is set to be slightly larger than the epidemic threshold ${\beta }_{th}\approx \langle k\rangle /\langle {k}^{2}\rangle$, where 〈*k*〉 and 〈*k*^2^〉 represent the average degree and the second order average degree^35^, respectively. Moreover, the recovery probability is *γ* = 0.3.

In order to characterize the spreading abilities of individuals, the spreading scope F(t) at time t is presented as the following:

5 $$F(t)=\frac{{n}_{I(t)}+{n}_{R(t)}}{n}$$

Where n is the number of individuals in a given network. Moreover, *n*_*I*(*t*)_ and *n*_*R*(*t*)_ represent the number of infected and recovered nodes at time t, respectively. It should be noted that all of the simulations are carried out on the real social networks. When all infected individuals are converted to the recovered state, the spreading process ends and the final spreading scope *F*(*t*_*c*_) is equal to the maximum values of the recovered individuals. Generally, within the same network, the larger the final spreading scope $F({t}_{c})$ triggered by initial spreaders, the stronger their spreading abilities.

## The Proposed Algorithm

From the previous section, it is resulted that a reasonable algorithm for the identification of the key individuals should focus on two aspects, including accuracy and simplicity. The accuracy is mainly reflected in the computational accuracy and stability, while simplicity is mainly correlated to the computational runtime. Especially for a social network, which usually contains millions of nodes, the key issue is how to reduce the complexity and improve the computational efficiency. In order to fill this gap, a novel algorithm is required to effectively reach a balance between the accuracy and the simplicity. According to Burt’s structural holes theory^36^, the individuals’ structural position in the social network is more important than the corresponding external relationship strength.Certain positional advantages indicate that the individuals occupying these positions have more information, resources and power than others. The positional advantages in the social network include local and global advantages as the following: The former advantages can be quantified by the local structural information^9,37^, while the latter advantages should consider global topological connections^8,38^. In this regard, it is intended to propose a three-step algorithm, named the CumulativeRank,in the present study. It is expected that the proposed algorithm can sufficiently combine node’s local and global performances. The details of the proposed algorithm are described as the following:

### Step 1:Quantifying the local advantage of each node

The structural hole theory provides a novel perspective for understanding the local performance of individuals. In fact, a structural hole is a gap between two unconnected nodes. When these two unconnected nodes are connected by a third node, the bridging node usually has more information advantages and control advantages since it acts as a mediator between different nodes. In order to quantify the control advantage of bridging nodes, Burt introduced the network constraint coefficient (NCC). The NCC for i^th^ node is described as the following:

6 $$NC{C}_{i}={\sum _{j\in \Gamma (i)}(p{}_{ij}+\mathop{\sum }\limits_{k=1,k\ne i,j}^{n}{p}_{ik}{p}_{kj})}^{2}$$

Where *p*_*ij*_ is the proportion of a given node *i*’s energy invested directly related to node *j*, which is written as the following:

7 $${p}_{ij}=\frac{{z}_{ij}}{\sum _{j\in \Gamma (i)}{z}_{ij}}$$

where *z*_*ij*_ is equal to 1 when a path from *i* to *j* exists, and it is zero otherwise. Γ(*i*) is the set of the nearest neighbours *i*. $\mathop{\sum }\limits_{k=1,k\ne i,j}^{N}{p}_{ik}{p}_{kj}$ measures the strength of the indirect connection from *i* to *j*. The NCC value of a node generally has a negative association with its influence in a given network. Therefore, it is found that as the NCC values reduce, the formation of structural holes is enhanced and subsequently the influence of nodes increases.

**Figure 1:**
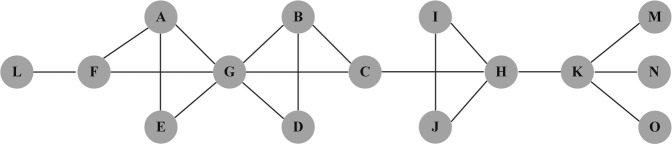
An example network consisted of 15 nodes and 19 edges.

Equation (6) indicates that the NCC value of a node is calculated based on its neighbourhood topology, including the number of neighbours and the the the corresponding closeness between them. However, the NCC has similar disadvantages to the DC. It only collects information of the nearest neighbours, while the structural information from farther neighbours is ignored. In fact, the NCC is ineffective when it faces the nodes bridging the same number of non-redundant contacts. For example, Fig. 1 illustrates that nodes C and F act as bridges between nodes G and H, and between nodes L and G, respectively. Based on Eq. (6), nodes C and F have the same value (i.e. NCCC = NCCF = 0.46). In other words, the two nodes have the same local influence. However, Fig. 1 shows that in addition to the common neighbour node G and its neighbours, node C has a high-order neighbour like node H, while node F only has one-order neighbour, which is entitled by node L. In fact, it is found that the spreading ability of each node highly depends on its neighbours^39^. For example, although nodes C and F have the same NCC values, node C has stronger spreading ability, which originates from wide range of contacts of high-order neighbours. Therefore, it is concluded that the NCC cannot accurately quantify the difference between nodes C and F in the abovementioned sample network.

This analysis shows that the NCC only collects information from the nearest neighbours, which leads to low resolution. In order to increase the accuracy of the method, more local structural information should be considered. Therefore, an improved network constraint coefficient (INCC) is proposed in the present study. It should be indicated that the INCC scheme is inspired by Chen’ semi-local centrality index^33^. In the proposed method, the local influence of a node combines the direct and indirect influences on its nearest and next nearest neighbours. Compared with the Burt’s NCC, INCC can provide richer connection information that individual has established, which gives a full understanding of the node influence in facilitating the information flow. The INCC value of node *i* is defined as the following:

8 $$INC{C}_{i}=\sum _{j\in \Gamma (i)}{({p^{\prime} }_{ij}+\mathop{\sum }\limits_{k=1,k\ne i,j}^{n}{p^{\prime} }_{ik}{p^{\prime} }_{kj})}^{2}$$

Where ${p^{\prime} }_{ij}=\frac{{Q}_{j}}{L{R}_{i}}$, *LR*_*i*_ and *Q*_*j*_ are calculated by the Eqs (1) and (2), respectively. Take node A as an example, $INC{C}_{A}={(\frac{{Q}_{F}}{L{R}_{A}}+\frac{{Q}_{G}}{L{R}_{A}}\times \frac{{Q}_{F}}{L{R}_{G}})}^{2}+{(\frac{{Q}_{G}}{L{R}_{A}}+\sum _{i\in (E,F)}\frac{{Q}_{i}}{L{R}_{A}})}^{2}+{(\frac{{Q}_{E}}{L{R}_{A}}+\frac{{Q}_{G}}{L{R}_{A}}\times \frac{{Q}_{E}}{L{R}_{G}})}^{2}$.

As shown in Fig. 1, node C has three nearest neighbours, including nodes B, G and H and seven next nearest neighbours, including nodes A, D, E, F, I, J and K, thus N_(C)_ = 10. The N values of the other nodes are presented in the third column of Table 1. According to Eqs (1) and (2), we can calculate Q_C_ = N_B_ + N_G_ + N_H_ = 24, LR_C_ = Q_B_ + Q_G_ + Q_H_ = 92. Similarly, Q_F_ = N_A_ + N_G_ + N_L_ = 18 and LR_F_ = Q_A_ + Q_G_ + Q_L_ = 71. Thus, it is easy to know the difference between node C and F by the corresponding INCC values, where INCC_C_ = 0.566 and INCC_F_ = 0.771. INCC_F_ > INCC_C_, that indicates node C has more spreading influence than node F. Table 1 shows that, node H has the lowest INCC value, which indicates that it has the largest local influence in the example network, while nodes L, M, N and O have the largest INCC values, indicating that they have the lowest influence. In a descending order of the nodal influence, the ranking result is H, G, K, C, F, B, A, D, E, I = J, L = M = N = O. It is found that the INCC can more accurately quantify the differences between node C and F, node A and B, node D and E, in comparison to the performance of the NCC method.

**Table 1:** The basic values of the example network.

i	DC_i_	N_i_	Q_i_	LR_i_	NCC_i_	INCC_i_	R_i_	CR_i_
A	3	7	21	76	0.676	0.886	15	0.168
B	3	7	24	82	0.676	0.829	15	0.164
C	3	10	24	92	0.460	0.566	4	0.055
D	2	6	15	67	0.785	0.928	15	0.172
E	2	6	15	64	0.785	0.937	15	0.173
F	3	7	18	71	0.460	0.771	7	0.096
G	6	8	43	117	0.384	0.328	5.5	0.047
H	4	9	25	71	0.406	0.323	3	0.027
I	2	4	13	38	0.953	0.994	15	0.177
J	2	4	13	38	0.953	0.994	15	0.177
K	4	7	21	46	0.25	0.365	3	0.031
L	1	3	7	18	1	1	15	0.178
M	1	4	7	21	1	1	15	0.178
N	1	4	7	21	1	1	15	0.178
O	1	4	7	21	1	1	15	0.178

### Step 2: Quantifying the global advantages of each node

Generally, the influential nodes also play a crucial role in maintaining the network connectivity. If these top influential nodes are removed or not involved in the spreading process, the final spreading scope and the spreading efficiency are reduced^40^. Consequently, the global performances of nodes are considered on maintaining the network connectivity and facilitating the information flow. In general, if the removal of a node leads to the network remaining more components and smaller connected components, the removed node is important in maintaining the network connectivity. The inherent attachment mechanism of social networks often leads to their excessive sensitivity to the removal of key nodes. In order to measure the vulnerability of a given network, Cozzens *et al*. proposed the concept of the tenacity^41^. As a vulnerability parameter of graph, the tenacity integrates three criteria, including the cost of the network breakage, the number of components and the size of largest connected component.

In this paper, the tenacity are introduced to assess the individual prominent position in maintaining the network connectivity. Due to the inhomogeneity of general networks, most nodes don’t belong to the cut-set of a given network, so random removal of these nodes cannot change the balance of the network structure or directly trigger the collapse of the network^42^. Thus, we redefine the tenacity of each node, which is denoted as the following:

9 $${R}_{i}=\,\min \{\frac{|i|+m(G-i)}{w(G-i)}:i\in V,w(G-i)\ge 2\}$$

**Figure 2:**
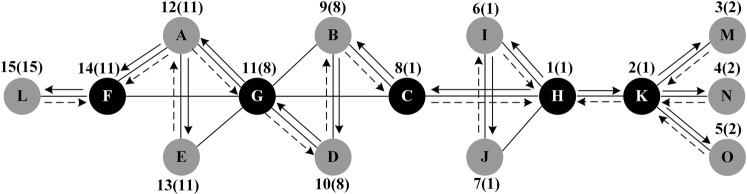
The identification process of key nodes in the example network: The solid arrowlines represent the forward edges, the dashed arrowlines represent the backtracking paths, the numbers above the nodes are their *dfn* values, and the numbers in brackets are their *low* values.

Where $|i|$ is the cost of node *i* removed, when the targeted removal obeys one by one model, $|i|=1$. $m(G-i)$ and $w(G-i)$ denote the giant component size after destruction and the number of components of the remaining network $G-i$. In Supplementary Note, an iterative algorithm is proposed for evaluating the nodes’ global performances. If node *i* plays a key role in maintaining the network connectivity, it either is the root with at least two descendants, or has a descendant *u* whose lowest depth-first number (*low*) is not less than its depth-first number (*dfn*). According to the above rule, we can identify five key nodes in the example network, which are H, G, K, C and F, respectively. The identification process is shown in Fig. 2.

### Step 3: Identifying the influential spreaders in the network

Based on the basic definitions mentioned above, a new ranking algorithm named the CumulativeRank algorithm is presented in this study, which measures the node spreading ability from two layers, including locally, the proposed algorithm considers both the local influence of nodes on their neighbours and globally, the algorithm considers the nodes’ prominent position in maintaining the network connectivity. The precise definition of the CumulativeRank is defined as the following:

10 $$C{R}_{(i)}=\frac{INC{C}_{i}}{\sqrt{\mathop{\sum }\limits_{j=1}^{N}INC{C}_{j}}}+\frac{T{C}_{i}}{\sqrt{\mathop{\sum }\limits_{j=1}^{N}T{C}_{j}}}$$

Where INCC_i_ is calculated by the Eq. (8). Moreover, *TC*_*i*_ is the normalized tenacity value of a given node *i*, $0\le T{C}_{i}\le 1$ and it is defined as the following:

11 $$T{C}_{i}=\frac{{R}_{i}-\underset{j=1}{\overset{N}{\min }}\{{R}_{j}\}}{\underset{j=1}{\overset{N}{\max }}\{{R}_{j}\}-\underset{j=1}{\overset{N}{\min }}\{{R}_{j}\}}$$

According to the above algorithm, nodes with the lowest CR values have the largest influence in facilitating information flow and maintaining the network connectivity. In a descending order of the nodal spreading ability, the ranking result of the example network is H, K, G, C, F, B, A, D, E, I = J, L = M = N = O.

## Experimental Evaluation

### Dataset

To validate the effectiveness of the proposed algorithm, the algorithm is evaluated in nine real social networks. All of the data except for OClinks can be downloaded from the Stanford network dataset^43^. Table 2 indicates that the six real social network include:(1) OClinks, which is a representative online community network, where users are from the University of California, Irvine^44^; (2)Ego-Facebook, which is an ego network consisting of friends lists from Facebook; (3)Soc-Epinions, which is a who-trust-whom online social network of a general consumer review site Epinions.com; (4)Wiki-Vote, which is a network containing all of the Wikipedia voting data from the inception of Wikipedia till January 2008; (5)Ca-HepPh, which is a collaboration network covering scientific collaborations between authors papers submitted to High Energy Physics from January 1993 to April 2003; (6) Email-Enron, which is an email communication network from Enron posted to the web by the Federal Energy Regulatory Commission during its investigation;(7) Ca-CondMat,which is a collaboration network covering scientific collaborations between authors papers submitted to Condense Matter category;(8) Ca-GrQc,which is a collaboration network covering scientific collaborations between authors papers submitted to General Relativity and Quantum Cosmology category;(9) Email-Eu-core,which is an email communication network from a large European research institution.

**Table 2:** The basic topological properties of the six social networks,including number of nodes *n* and edges *m* within the networks, average degree 〈*k*〉, characteristic path length *L*, clustering coefficient *C*, degree heterogeneity $H=\langle {k}^{2}\rangle /{\langle k\rangle }^{2}$, epidemic threshold ${\beta }_{th}\approx \langle k\rangle /\langle {k}^{2}\rangle$.

Network	n	m	〈k〉	L	C	H	β_th_
OClinks	1899	20296	21.375	3.197	0.085	3.773	0.012
Ego-Facebook	4039	88234	43.691	3.693	0.606	2.439	0.009
Soc-Epinions	75879	508837	6.706	11.549	0.138	16.569	0.009
Wiki-Vote	7115	103689	14.573	3.341	0.141	9.803	0.007
Ca-HepPh	12008	118521	19.74	4.673	0.612	28.21	0.002
Email-Enron	36692	183831	10.732	4.025	0.497	13.265	0.007
Ca-CondMat	23133	93497	8.0835	5.352	0.706	2.734	0.045
Ca-GrQc	5242	14496	5.531	6.049	0.687	3.049	0.059
Email-Eu-core	1005	25571	33.246	2.653	0.372	5.614	0.005

### Experimental results

In general, the super-spreader in a social network is regarded as an individual that has greater spreading abilities. In other words, it is capable of widely spreading the message to other recipient individuals infinitely. In this section, the standard SIR model is utilized to simulate the spreading processes in real social networks.

The ranking order of each node is calculated in the above mentioned six networks initially according to DC, BC, EC, LR, PR, FA and CR, respectively. In a sample network, if nodes have the same calculated scores according to the same ranking algorithm, they will have the same rank. In the present study, we are more interested in the super-spreaders instead of all nodes in the network. Thus, only the top nodes of the ranking list are considered. For this purpose, the top-10 nodes of each ranking algorithm are selected as the initial spreaders and all of the other nodes in the network are marked as susceptible nodes.

Table 3 shows that the ranking results of the proposed algorithm are quite different from those of other ranking algorithms. In Wiki-Vote, it is observed that the proposed algorithm suggests node 326 as the most influential node, followed by the nodes 656 and in the third position comes node 488. The same data is not found for the other ranking algorithms, indeed DC, BC and LR suggest node 699 as the most influential node, followed by nodes 286 and 2374 (third for DC and LR) or 409(third for BC). It is indicated that the CR gives the ranking results with the most significant difference from other five well-known ranking algorithms in Ca-HepPh and Ca-CondMat, as none of the 10 key nodes identified by CR appear in the top 10 nodes for DC, BC, EC, PR and LR. In addition, it is observed that both the proposed algorithm and the FA algorithm combine the global and local attributes of the network, however there are great differences between the two algorithms in terms of ranking results.

**Table 3:** The top-10 ranked nodes by the proposed method and their corresponding ranks by degree centrality (DC), betweenness centrality (BC), eigenvector centrality (EC), LocalRank (LR), PageRank (PR),Fu *et al*.’ algorithm (FA) and CumulativeRank(CR).

OClinks								Ego-Facebook								Soc-Epinions							
Rank	DC	BC	EC	LR	PR	FA	CR	Rank	DC	BC	EC	LR	PR	AF	CR	Rank	DC	BC	EC	LR	PR	FA	CR
1	444	35	444	49	35	443	718	1	0	107	1912	107	3437	106	3437	1	363	14	14	14	14	13	1677
2	35	718	49	4	718	34	35	2	107	1684	2347	1912	107	1911	107	2	1677	1677	363	363	1677	362	14
3	49	49	4	444	444	48	49	3	1684	3437	2266	2347	1684	1683	1684	3	14	530	1867	40	1867	1676	2776
4	718	4	35	35	49	717	6	4	1912	1912	2233	2543	0	3436	1912	4	1867	363	40	1867	363	1866	1867
5	4	444	64	336	4	3	635	5	3437	1085	2543	1888	1912	4038	0	5	2776	1867	530	530	8499	529	8499
6	38	6	718	331	6	37	4	6	2543	0	2206	1800	348	1887	3980	6	40	40	185	185	530	184	363
7	2	2	336	2	2	1	2	7	2347	698	1985	1663	686	1799	414	7	530	2776	23	23	230	229	4125
8	6	38	2	343	38	5	232	8	1888	567	2142	1352	3980	1662	348	8	23	230	230	150	2776	39	563
9	64	635	38	64	64	63	20	9	1800	58	2464	1431	414	1351	686	9	185	8499	132	230	185	131	530
10	336	64	57	57	336	335	54	10	1663	428	2218	1199	483	1729	698	10	30	185	150	132	40	322	185
**Wiki-Vote**								**Ca-HepPh**								**Email-Enron**							
**Rank**	**DC**	**BC**	**EC**	**LR**	**PR**	**FA**	**CR**	**Rank**	**DC**	**BC**	**EC**	**LR**	**PR**	**FA**	**CR**	**Rank**	**DC**	**BC**	**EC**	**LR**	**PR**	**FA**	**CR**
1	699	699	905	699	326	698	326	1	363	279	297	297	363	362	1056	1	271	271	79	79	271	143	144
2	286	286	326	286	409	2373	656	2	297	827	328	328	297	296	564	2	144	80	85	182	144	270	80
3	2374	409	409	2374	1332	285	488	3	328	472	518	518	328	327	788	3	191	92	182	85	80	190	92
4	408	410	711	1052	656	407	686	4	440	509	611	611	440	439	976	4	80	93	245	245	191	196	191
5	1052	902	666	2013	905	2012	409	5	518	475	648	363	518	517	464	5	197	148	144	197	93	79	197
6	2013	1052	286	3712	711	1051	1224	6	327	890	545	327	327	610	4549	6	85	144	197	144	92	84	148
7	3712	1146	626	408	686	3711	177	7	611	295	327	648	611	326	1284	7	245	191	95	95	197	244	245
8	517	666	1141	2	699	516	1374	8	507	528	440	545	507	506	871	8	148	46	89	440	148	147	244
9	483	656	1005	517	2	482	926	9	586	724	363	440	586	585	2157	9	79	197	1233	89	245	78	85
10	1221	7	247	483	666	1220	1141	10	326	1619	507	507	326	325	879	10	92	1233	191	1233	1268	91	93
**Ca-CondMat**								**Ca-GrQc**								**Email-Eu-core**							
**Rank**	**DC**	**BC**	**EC**	**LR**	**PR**	**FA**	**CR**	**Rank**	**DC**	**BC**	**EC**	**LR**	**PR**	**FA**	**CR**	**Rank**	**DC**	**BC**	**EC**	**LR**	**PR**	**FA**	**CR**
1	349	349	949	949	349	853	2135	1	101	101	1037	101	108	1036	108	1	160	160	160	160	160	159	377
2	949	949	3880	3880	949	259	1265	2	295	279	11	279	1037	185	2138	2	121	86	107	121	62	120	5
3	3073	3073	2092	2092	1184	438	9960	3	279	265	207	265	577	1031	11	3	107	5	62	82	86	81	211
4	3880	1369	349	349	260	1038	11441	4	103	77	53	77	295	107	1731	4	62	121	434	107	107	106	107
5	2092	854	2116	255	1369	348	774	5	77	296	577	72	11	10	1137	5	86	62	121	62	121	85	462
6	1369	41	3073	2116	41	2134	2172	6	72	159	20	288	186	52	53	6	82	107	183	434	129	61	971
7	1517	1184	255	3073	3073	2171	527	7	296	288	147	296	103	30	1118	7	434	64	128	249	183	433	121
8	255	260	4068	947	1057	1033	5801	8	288	295	186	103	101	1732	315	8	183	82	129	86	5	165	411
9	41	3880	1637	41	527	768	439	9	265	285	108	159	53	206	20	9	5	377	256	183	434	182	560
10	947	1057	2119	585	255	3579	71	10	100	302	288	295	1733	364	123	10	129	129	249	166	64	63	495

**Figure 3:**
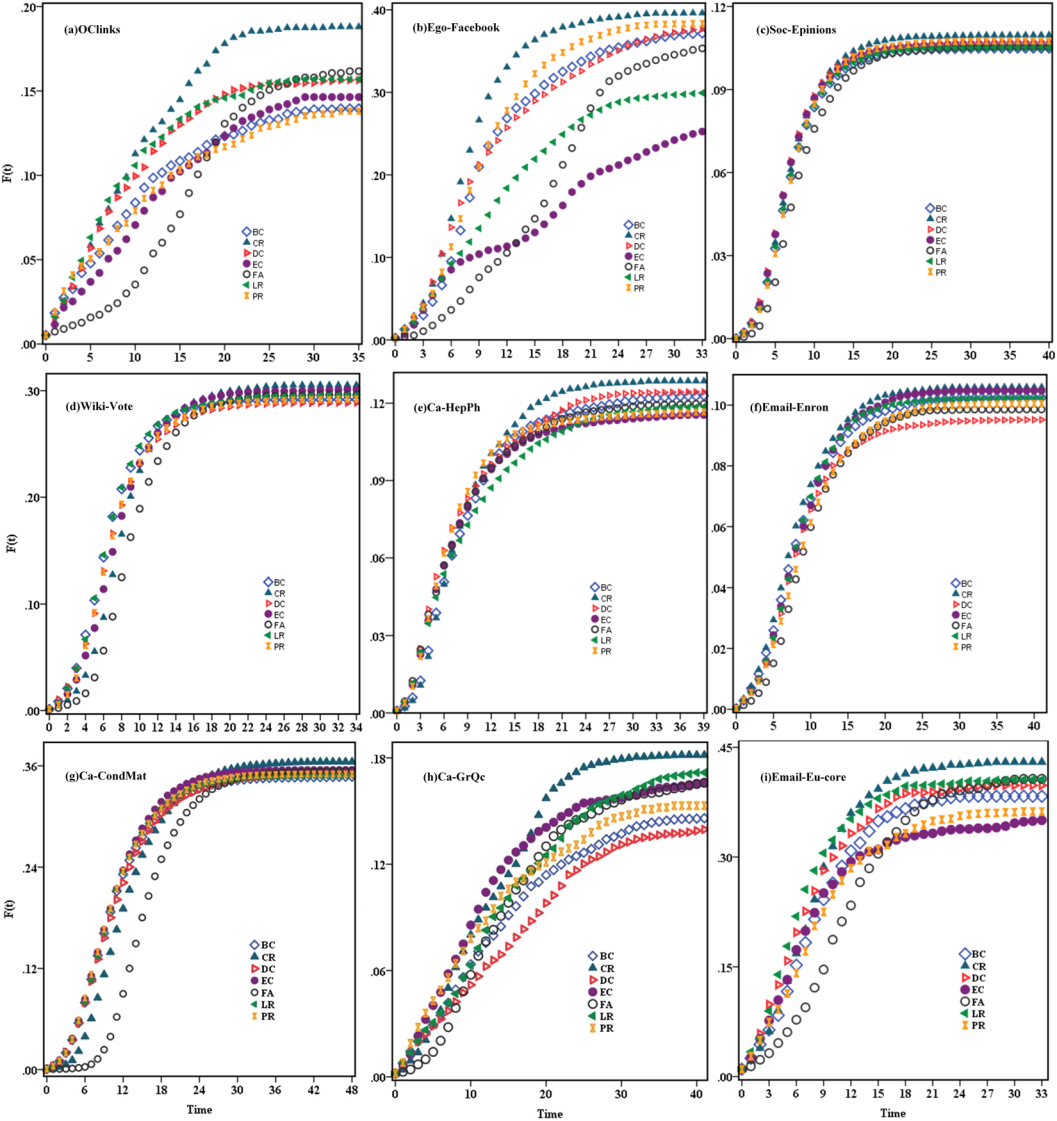
Plot of the spreading scope of the top-10 nodes ranked by different ranking algorithms in (**a**) OClinks, (**b**)Ego-Facebook, (**c**) Soc-Epinions, (**d**)Wiki-Vote, (**e**) Ca-HepPh, (**f**) Email-Enron, (**g**) Ca-CondMat, (**h**) Ca-GrQc and (**i**) Email-Eu-core. The infection probability *β* are 0.02 in (**a**), 0.01 in (**b**–**f**,**i**), 0.05 in (**g**) and 0.06 in (**h**), the recovery rate *γ* is 0.3. The results are averaged over 100 independent runs.

In order to evaluate the performances of the proposed algorithm, the all-contact SIR model is applied to simulate the information spreading process. In the all-contact SIR model each infected node can contact all of its susceptible neighbours at per time step. Figure 3 shows the simulation result of the spreading scopes, F (t), as a function of time for nine networks. For each initial infective node set, the SIR process is repeated 100 times to ensure the stability of the results (see the Supplementary Note). By comparing the changes of F (t) under different spreading sources, it is observed that in almost all of the networks, the initial spreaders obtained by the CR algorithm spread the information faster and the final spreading scopes *F*(*t*_*c*_) always reach the highest value (see Table 4). In the cases of OClinks, Ego-Facebook, Ca-HepPh,Ca-GrQc and Email-Eu-core, it is observed that the performance of the CR is much better than those of other algorithms. Even though in Soc-Epinions, the initial infection sources determined by different ranking algorithms are very similar and the changes of F (t) under different spreading sources perform nearly the same, but the proposed algorithm is slightly better and the final spreading scope still reaches the highest value. Relatively, other six classical ranking algorithms do not show significant consistency in the change tendency of F (t). Although the performance of the EC can outperform the other five algorithms except the CR in Wiki-Vote and Email-Enron, DC and LR perform better than EC in OClinks, Ego-Facebook, Ca-HepPh and Email-Eu-core. Experimental results show that the ranking of the proposed algorithm is more accurate and stable.

**Table 4:** The final spreading scopes *F*(*t*_*c*_) for the nine real networks under different ranking algorithms. n is the total number of nodes, *P* is the ratio of the number of source spreaders and *β* is the infection rate.

Datasets	Algorithms	Final spreading scopes	Time steps	n	*β*	*P*
OClinks	DC	0.157	39	1899	0.02	0.0053
	BC	0.14	35			
	EC	0.146	35			
	LR	0.157	31			
	PR	0.138	42			
	FA	0.163	40			
	**CR**	**0.188**	**36**			
Ego-Facebook	DC	0.383	54	4039	0.01	0.0025
	BC	0.381	52			
	EC	0.298	58			
	LR	0.302	51			
	PR	0.384	40			
	FA	0.374	56			
	**CR**	**0.396**	**33**			
Soc-Epinions	DC	0.107	41	75879	0.01	0.0001
	BC	0.105	46			
	EC	0.106	42			
	LR	0.105	43			
	PR	0.108	41			
	FA	0.105	43			
	**CR**	**0.110**	**40**			
Wiki-Vote	DC	0.288	35	7115	0.01	0.0014
	BC	0.292	45			
	EC	0.301	42			
	LR	0.296	42			
	PR	0.292	41			
	FA	0.296	36			
	**CR**	**0.305**	**34**			
Ca-HepPh	DC	0.124	41	12008	0.01	0.0008
	BC	0.123	61			
	EC	0.115	53			
	LR	0.119	44			
	PR	0.119	55			
	FA	0.120	40			
	**CR**	**0.129**	**39**			
Email-Enron	DC	0.095	45	36692	0.01	0.0003
	BC	0.102	43			
	EC	0.105	40			
	LR	0.102	44			
	PR	0.101	46			
	FA	0.099	44			
	**CR**	**0.106**	**41**			
Ca-CondMat	DC	0.355	66	23133	0.05	0.0004
	BC	0.0.47	56			
	EC	0.355	48			
	LR	0.349	66			
	PR	0.350	63			
	FA	0.355	55			
	**CR**	**0.365**	**48**			
Ca-GrQc	DC	0.141	49	5242	0.06	0.0019
	BC	0.146	48			
	EC	0.167	48			
	LR	0.172	51			
	PR	0.153	45			
	FA	0.168	51			
	CR	0.182	41			
Email-Eu-core	DC	0.398	34	1005	0.01	0.01
	BC	0.383	35			
	EC	0.357	41			
	LR	0.406	37			
	PR	0.362	40			
	FA	0.407	37			
	**CR**	**0.430**	**33**			

The SIR model is based on discrete step iterations to demonstrate the spreading process of information. If information cannot continue to flow between nodes according to the model rules, iterations end and the network reaches its stable state. The final time required for the end of the spreading process is obtained from the number of iterations. In general, the higher the number of iterations, the longer information propagates within a given network. Table 4 shows that in the same network, the difference in the spreading sources can increase the number of iterations and prolong the time of information propagation in reaching a steady state. For example, in Ego-Facebook, when β = 0.01, the spreading sources selected by the CR have the least number of iterations before convergence and the time steps are 33. The PR performs next above the CR, and then LR, BC, DC and EC show ascending order of the number of iterations. In contrast, the CR has the average lesser number of iterations before convergence, however it achieves the largest final spreading scope in all sample networks, which means that the spreading sources selected by the CR effectively accelerate the spreading process of information.

The total runtime of the CR consists of two parts, including the time of computing INCC values for all nodes and the time of quantifying the tenacity of each node. For the former, as *N*_*w*_ requires traversing node *v*’s neighbourhood within two steps and takes *O*(〈*k*〉^2^) time on average, the time complexity is *O*(*n*〈*k*〉^2^), where *n* and 〈*k*〉 are the total number of nodes and the average degree in a given network, respectively. After each iteration, the number of components $w(G-i)$ and the size of the largest connected component $m(G-i)$ are re-calculated, which takes the complexity *O*(*n*^2^). Totally, the whole time complexity of the proposed algorithm is *O*(*n*〈*k*〉^2^ + *n*^2^). In contrast, the time complexity of the CR is much lower than that of the BC and the EC, which have the complexity of $O({n}^{2}\,\log \,n+nm)$ and *O*(*n*^3^) respectively. These are close to the PR with the complexity of *O*(*n*^2^ + *n*). Analysis of time complexity demonstrates that the proposed algorithm has relatively less computational burden in identifying potential super-spreaders and can be applicable to large-scale networks.

## Conclusion

In the present study, a three-step ranking algorithm, named the CumulativeRank, is proposed in order to identify and quantify potential super-spreaders in a social network. Previous studies have shown that the nodal spreading ability originate from the local prominent position and the total connectivity strength that the node obtains. The proposed algorithm sufficiently combines the node’s local and global performances. Locally, inspired by Burt’s structural holes theory, the improved network constraint coefficient is proposed based on the semi-local centrality index. Compared with the conventional network constraint coefficient, the improved network constraint coefficient provides richer connection information for evaluating the local performance of each node. Globally, the concept of the tenacity are introduced to evaluate the nodes’ global connectivity strengths. Furthermore, extensive experiments on real-world social networks show explicitly that the proposed algorithm outperforms the existing well-known ranking algorithms and can be applicable to large-scale networks.

## Supplementary information

Supplementary information

Supplementsry Result
