# Supplementary material for: Identifying and quantifying potential super-spreaders in social networks
Source: Sci Rep. 2019 Oct 15;9:14811. doi: 10.1038/s41598-019-51153-5 (PMC6794301; doi:10.1038/s41598-019-51153-5)
Supplement: Supplementary file 1 — Supplementary information [file 41598_2019_51153_MOESM1_ESM.docx]

Identifying and quantifying potential super-spreaders in social networks

Dayong Zhang^a^,Yang Wang^b^, Zhaoxin Zhang^b*^

^a^ Department of New Media and Arts, Harbin Institute of Technology, Harbin150001,China

^b^School of Computer Science and Technology, Harbin Institute of Technology , Weihai 264209,China

Corresponding author. Tel.: +86 451 86404608.

E-mail address:heart@hit.edu.cn (Z.-X. Zhang).

1、Implementation process of the improved network constraint coefficient (INCC)

# encoding: utf-8

import pickle

'''这个脚本是约束二的指标'''

'''

因为给的数据id不是从0开始的所以,自己处理一下,重新将id和数量对应起来

'''

def prepareName_name():

fp = open("data\\newdata\\2220\\2220Nodes.txt",'r')

fp1 = open("data\\newdata\\2220\\name_number.txt",'w')

i=-1

j=0

for line in fp.readlines():

# print line

i+=1

if i==0:

continue

line = line.strip()

data = line.split(",")

id = int(data[0])

fp1.write(str(j)+","+str(id)+"\n")

j+=1

fp.close()

fp1.close()

def prepareNumber_number():

fp = open("data\\newdata\\2220\\2220Edges.txt",'r')

fp1 = open("data\\newdata\\2220\\name_number.txt",'r')

fp2 = open("data\\newdata\\2220\\number_number.txt",'w')

name_number = {}

for line in fp1.readlines():

line = line.strip()

data = line.split(",")

id = int(data[0])

name = int(data[1])

name_number[name] = id

for item in name_number.iteritems():

print str(item[0])+","+str(item[1])

for line in fp.readlines():

line = line.strip()

data = line.split(",")

source = int(data[0])

target = int(data[1])

source_ = name_number.get(source)

target_ = name_number.get(target)

fp2.write(str(source_)+","+str(target_)+"\n")

fp.close()

fp1.close()

# prepareName_name()

# prepareNumber_number()

def getname_number():

name_number = {}

fp = open("data\\gouzaoshuju\\name_number.txt",'r')

for line in fp.readlines():

line = line.strip()

data = line.split(",")

name_number[str(data[0])]=data[1]

fp.close()

return name_number

def getTarget():

#节点的个数

N = 1893

#分别初始化好各个指标的存储空间

n = [0 for i in range(N)]

res = [0 for i in range(N)]

Q = [0 for i in range(N)]

C = [0 for i in range(N)]

m = [[] for i in range(N)]

# fp = open("data\\newdata2018-10-22\\gemsec_facebook_dataset\\tvshow_edges.csv",'r')

# fp = open("data\\newdata\\2220\\number_number.txt",'r')

# fp = open("data\\Twitter mentions and retweets_\\number_number.txt",'r')

fp = open("data\\data_2019_1_11\\OClink\\1893-node_number_number.txt",'r')

for line in fp.readlines():

datas = line.split(",")

num1 = int(datas[0])

num2 = int(datas[1])

#不能重复添加元素，这里考虑的只是网络，并没有考虑权重的问题，所以可以这样写

if num2 not in m[num1]:

m[num1].append(num2)

#在这处理异步,按无向图处理,就不用处理源文件了(源文件中1,0 但是没有0,1这一项)

if num1 not in m[num2]:

m[num2].append(num1)

fp.close()

#计算N

for i in range(N):

tempn = []

for j in m[i]:

tempn.append(j)

if i in m[j]:

tempn.extend(m[j])

tempn.remove(i)

continue

tempn.extend(m[j])

#这里去掉重复的元素了

tempn = list(set(tempn))

n[i] = len(tempn)

#计算Q和C

#当前节点是i

for i in range(N):

#遍历i节点的每个直接相邻节点

q = 0.0

for j in m[i]:

q+=n[j]

Q[i] = q

#当前节点是i,在word中的公式表示是有错误的,,要理解一下

for i in range(N):

#遍历i节点的每个直接相邻节点

c = 0.0

for j in m[i]:

c+=Q[j]

C[i] = c

#计算结果

#当前节点是i,看公式确定pij是什么,下面的temp相当于是公式小括号里面的值

for i in range(N):

#遍历i节点的每个直接相邻节点

temp = 0.0

for j in m[i]:

#转换成浮点数来计算

temp+=(float)(Q[j])/(float)(C[i])#(这里是pij有的'的没办法描述而已)

#检查均相邻的节点

for k in m[j]:

if i in m[k]:

temp+=(Q[k]/C[i])*(Q[j]/C[k])

res[i] += temp*temp

temp = 0.0

# if res[i]==0.0:

# print "有res是0,节点标号"+str(i)

table = {}

for i in range(len(res)):

table[i] = res[i]

'''升序排序,这里其实就相当于是按照过程二重新标号'''

t = sorted(table.items(),key = lambda x:x[1],reverse = False)

# name_number = getname_number()

# for item in name_number.items():

# print item[0]+":"+item[1]

#将产生的结果写入文件中把,每次计算的时候都太费时间了

f = open("data\\data_2019_1_11\\OClink\\1893-node_ED.txt",'w')

result = {}

'''排序前十的节点'''

for i in range(N):

# if str(t[i][0])=='0':

# continue

# print name_number[str(t[i][0])]+" " +str(t[i][1])

# print str(t[i][0])+" " +str(t[i][1])

result[str(t[i][0])] = float(t[i][1])

f.write(str(t[i][0])+","+str(t[i][1])+"\n")

f.close()

return result

if __name__=="__main__":

result = getTarget()

# for key in result.keys():

# print str(key)+":"+str(result[key])

**************************************************************************

2、Iterative algorithm for evaluating the global performances.

______________________

# encoding: utf-8

import copy

import pr1

import math

import time

N = 1893

next = 0 # Next index.

index = [None] * N

lowlink = [None] * N

onstack = [False] * N

stack = []

nextgroup = 0 # Next SCC ID.

groups = [] # SCCs: list of vertices.

groupid = {} # Map from vertex to SCC ID.

adj = [[] for i in range(N)]

father = [None] * N

'''

删除一个无向图中的点，能使得原图增加几个连通分量？

如果该点是一个孤立的点，那么增加-1个。

如果该点不是割点，那么增加0个。

如果该点是割点且非根节点，那么增加该点在dfs树中(无反向边连回早期祖先的)的儿子数。

如果该点是割点且是一个dfs树的根节点，那么增加该点在dfs树中(无反向边连回早期祖先的)的儿子数-1的数目，也就是增加了以该dfs树的儿子数目-1

'''

#储存的是删除该给点后增加的连通分量的数目

cut = [0] * N

#表示每个节点是否为割点

isCut = [False] * N

#存储孩子节点的个数

child = [0] * N

def init():

fp = open("data\\data_2019_1_11\\OClink\\1893-node_number_number.txt",'r')

for line in fp.readlines():

datas = line.split(",")

num1 = int(datas[0])

num2 = int(datas[1])

#不能重复添加元素，这里考虑的只是网络，并没有考虑权重的问题，所以可以这样写

if num2 not in adj[num1]:

adj[num1].append(num2)

#在这处理一步,按无向图处理,就不用处理源文件了(源文件中1,0 但是没有0,1这一项)

if num1 not in adj[num2]:

adj[num2].append(num1)

# Tarjan's algorithm, iterative version.

def sconnect(v,adj):

global next, nextgroup

work = [(v, 0)] # NEW: Recursion stack.

while work:

v, i = work[-1] # i is next successor to process.

# print "迭代的节点,其中在(a,b)中a代表节点,b代表节点是第几次访问"+str(work)

del work[-1]

#如果v是第一次访问

if i == 0: # When first visiting a vertex:

index[v] = next

lowlink[v] = next

next += 1

stack.append(v)

# print "栈中的节点"+str(stack)

onstack[v] = True

recurse = False

for j in range(i, len(adj[v])):

#搜索下一个邻接点

w = adj[v][j]

#邻接点没有访问过

if index[w] == None:

#没有访问过的节点才算是孩子节点

child[v] = child[v]+1

# 入栈,标记之后要访问的节点,记录父节点

father[w]=v

#孩子节点的个数加一

work.append((v, j+1))

work.append((w, 0))

recurse = True

break

#邻接点已经访问过

elif onstack[w]:

lowlink[v] = min(lowlink[v], index[w])

#recurse为False表示节点的所有子节点已经访问过

if recurse: continue

#如果v是一个根节点,将根以及所有子节点退栈

if index[v] == lowlink[v]:

com = []

while True:

w = stack[-1]

del stack[-1]

onstack[w] = False

com.append(w)

groupid[w] = nextgroup

if w == v: break

groups.append(com)

nextgroup += 1

#在递归的过程时递归一个节点就更新一次,虽然在迭代的时候只更新一次但是因为放入了栈中,

#栈不空就更新,所以最后会全部更新

if work:

w = v

v, _ = work[-1]

lowlink[v] = min(lowlink[v], lowlink[w])

def run(exclude,adj):

for v in xrange(N):

# print v

if v in exclude:

continue

if index[v] == None:

sconnect(v,adj)

def res():

for i in range(len(father)):

if father[i] == None:

continue

if lowlink[i]>=index[father[i]]:

# print "i="+str(i)+"--节点"+str(father[i])+"是割点"

cut[father[i]] = cut[father[i]]+1

isCut[father[i]]=True

'''

从数据中删除割点,即把ajd中割点对应的位置置为[],而且把每个子list中含有的相应的元素删除

'''

def deleteNode(num):

global adj,next,index,lowlink,lowlink,onstack,stack,nextgroup,groups,groupid,father,cut,isCut,child,N

#把数据复制一份,避免污染原始数据

newadj = copy.deepcopy(adj)

newadj[num] = []

for item in newadj:

if num in item:

item.remove(num)

next = 0

index = [None] * N

lowlink = [None] * N

onstack = [False] * N

stack = []

nextgroup = 0 # Next SCC ID.

groups = [] # SCCs: list of vertices.

groupid = {} # Map from vertex to SCC ID.

father = [None] * N

cut = [0] * N

isCut = [False] * N

child = [0] * N

#将删除割点之后的数据返回

return newadj

'''

遍历每个割点求相应的MGS的值

MGS即为删除S点后网络中的最大连通分支包含的节点的数目

'''

def getMGS_WGS(nodes):

MGS = {}

WGS = {}

t=0

for item in nodes:

t=t+1

if t%500==0:

print "tarjan算法运行了"+str(t)+"个节点了"

# print "删除一个节点之后再一次运行程序*************"

newadj = deleteNode(item)

#删除割点之后在一次运行程序

run([item],newadj)

res()

# print "groups:"+str(groups)

# print "lowlink:"+str(lowlink)

# print "father:"+str(father)

# print "index:"+str(index)

# print "isCut:"+str(isCut)

#如果是根节点还要额外的判断一下

# print "cut:"+str(cut)

k=0

for i in range(len(groups)):

if k<len(groups[i]):

k = len(groups[i])

MGS[item]=k

WGS[item]=len(groups)

return MGS,WGS

def computeTGS(MGS,WGS):

TGS = {}

KEY = list(MGS)

MGS_VALUE = list(MGS.values())

WGS_VALUE = list(WGS.values())

for i in range(len(MGS)):

s = 0

s = s+1+int(MGS_VALUE[i])

s = (float)(s)/(float)(WGS_VALUE[i])

TGS[KEY[i]] = s

return TGS

def getAllGTS(index, lowlink, groups, adj, i, father, cut, isCut):

global N

start = time.time()

#先调用一下初始化的方法

init()

run([],adj)

res()

end = time.time()

print "tarjan运行一遍的时间为:"+str(end-start)+"秒"

# print "groups:" + str(groups)

# print "lowlink:" + str(lowlink)

# print "father:" + str(father)

# print "index:" + str(index)

# print "isCut:" + str(isCut)

# #如果是根节点还要额外的判断一下

# print "cut:" + str(cut)

# print adj

geDians = []

for i in range(len(isCut)):

if isCut[i] == True:

geDians.append(i)

fp = open("data\\data_2019_1_11\\OClink\\1893-node_gedian.txt",'w')

for i in geDians:

fp.write(str(i)+"\n")

fp.close()

#获取所有节点

nodes = []

nodes.extend(geDians)

for i in range(N):

if i not in geDians:

nodes.append(i)

MGS, WGS = getMGS_WGS(nodes)

# print MGS

# print WGS

TGS = computeTGS(MGS, WGS)

fp = open("data\\data_2019_1_11\\OClink\\1893-node_TGS.txt",'w')

for key in TGS.keys():

fp.write(str(key)+","+str(TGS[key])+"\n")

fp.close()

# print "---------------"

# print TGS

#下面是的输出对应的ri的值

# name_number = pr1.getname_number()

# for key in TGS:

# print name_number[str(key)]+" " +str(TGS[key])

return TGS

def computeRi(TGS):

Ri = {}

minri = 1000000

maxri = -1

for key in TGS.keys():

ri = TGS[key]

if minri>ri:

minri = ri

if maxri<ri:

maxri = ri

for key in TGS.keys():

ri = TGS[key]

temp = (float)(ri-minri)/(float)(maxri-minri)

Ri[key] = temp

fp = open("data\\data_2019_1_11\\OClink\\1893-node_Ri.txt",'w')

for key in Ri.keys():

fp.write(str(key)+","+str(Ri[key])+"\n")

fp.close()

# name_number = pr1.getname_number()

# for key in TGS:

# print name_number[str(key)]+" " +str(Ri[key])

return Ri

def computeTi(ED,Ri):

totalRi=0

for key in Ri:

totalRi = totalRi+Ri[key]

totalED = 0

for key in ED:

totalED = totalED+ED[key]

print "totalRi:"+str(totalRi)

print "totalED:"+str(totalED)

Ti = {}

for key in Ri:

EDi = ED[str(key)]

ri = Ri[key]

# print key

# print EDi

# print ri

temp1 = (float)(EDi)/(float)(math.sqrt(totalED))

temp2 = (float)(ri)/(float)(math.sqrt(totalRi))

Ti[key] = temp1+temp2

print "%%%%%%%%%%%%%%%%%%%%%%%%%%"

fp = open("data\\data_2019_1_11\\OClink\\1893-node_Ti.txt",'w')

for key in Ti.keys():

fp.write(str(key)+","+str(Ti[key])+"\n")

fp.close()

return Ti

def getED():

ED = {}

fp = open("data\\data_2019_1_11\\OClink\\1893-node_ED.txt",'r')

for line in fp.readlines():

line = line.strip()

datas = line.split(",")

num1 = str(datas[0])

num2 = float(datas[1])

ED[num1] = num2

return ED

def getRi():

Ri = {}

fp = open("data\\data_2018-12-26\\soc-Epinions1_Ri.txt",'r')

for line in fp.readlines():

line = line.strip()

datas = line.split(",")

num1 = str(datas[0])

num2 = float(datas[1])

Ri[num1] = num2

fp.close()

return Ri

def Ti_paixu():

fp = open("data\\data_2019_1_11\\OClink\\1893-node_Ti.txt","r")

table = {}

for line in fp.readlines():

line = line.strip()

datas = line.split(",")

table[int(datas[0])]=float(datas[1])

fp.close()

t = sorted(table.items(),key = lambda x:x[1],reverse = False)

fp = open("data\\data_2019_1_11\\OClink\\1893-node_Ti_paixu.txt","w")

for i in range(N):

fp.write(str(t[i][0])+","+str(t[i][1])+"\n")

fp.close()

if __name__=="__main__":

start = time.time()

TGS = getAllGTS(index, lowlink, groups, adj, i, father, cut, isCut)

print "函数getAllGTS运行完成"

Ri = computeRi(TGS)

# Ri = getRi()

print "函数computeRi运行完成"

# ED = pr1.getTarget()

ED = getED()

print "函数getED运行完成"

Ti = computeTi(ED,Ri)

print "函数computeTi运行完成"

end = time.time()

print "花费的时间为:"+str(end-start)+"秒"

Ti_paixu()
